# Prognostic Impact of *BRCA1* and *BRCA2* Mutations on Long-Term Survival Outcomes in Egyptian Female Breast Cancer Patients

**DOI:** 10.3390/biology10070566

**Published:** 2021-06-22

**Authors:** Sherihan AbdelHamid, Hala El-Mesallamy, Hany Abdel Aziz, Abdel-Rahman Zekri

**Affiliations:** 1 Department of Biochemistry, Faculty of Pharmacy, Ain Shams University, Cairo 11566, Egypt; hala.elmosalamy@pharma.asu.edu.eg; 2 Dean of Faculty of Pharmacy, Sinai University, North Sinai 45518, Egypt; 3 Department of Clinical Oncology, Faculty of Medicine, Ain Shams University, Cairo 11591, Egypt; hany.kasem@med.asu.edu.eg; 4 Virology and Immunology Unit, Cancer Biology Department, National Cancer Institute, Cairo University, Giza 11796, Egypt; abdelrhman.zekri@nci.cu.edu.eg

**Keywords:** *BRCA1/2* mutations, clinical outcome, breast cancer, prognosis, Egypt

## Abstract

**Simple Summary:**

Countries with emerging economies suffer from a high incidence of breast cancer and advanced stage at diagnosis, coupled with limited health and medical care resources. Egypt has witnessed the world’s oldest documented cancer case, more than 3500 years BC, and the Egyptian population shows a high degree of genetic diversity compared to other populations due to its complex and diverse ethnic origins, with high incidence and mortality rates of breast cancer. Though the incidence and profile of BRCA1/2 mutations is population specific, data on population-based clinical outcomes are scarce. In this context, this study is an attempt to elucidate the long-term prognostic implications of BRCA1/2 mutations in Egyptian female breast cancer patients over 24 years. We believe that our findings provide indicators to implement screening strategies as well as optimize treatment options and prophylactic measures for BRCA1/2 carriers that can be applied in the routine clinical practice.

**Abstract:**

Evidence on the prognostic relevance of *BRCA1/2* mutations on breast cancer survival is still debatable. To address this ambiguity, we sought to elucidate the impact of *BRCA1*/*2* mutation carriership on long-term clinical outcomes for the first time in Egyptian female breast cancer patients. This study comprised 103 Egyptian female breast cancer patients previously tested for *BRCA1/2* mutations. Clinicopathological characteristics and long-term follow-up data were retrieved from clinical records until death or loss to follow-up. Overall survival (OS), recurrence-free survival (RFS), disease-free survival (DFS), and metastasis-free survival (MFS) were compared in *BRCA1/2* mutation carriers and non-carriers. Pathogenic variants (Class 5 according to ACMG/AMP guidelines) were observed in 29 cases. The profile of the detected variants was previously reported. After median follow-up time of 6.9 years (range, 4.2–24.4 years), *BRCA1/2* carriers exhibited significantly worse RFS compared to non-carriers (*p* = 0.01; HR = 3.00 (95%CI 1.35–6.68)). However, we couldn’t demonstrate statistically significant difference between carriers of pathogenic mutations and non-carriers regarding MFS (*p* = 0.24; HR = 1.38 (95%CI 0.8–2.4)), DFS (*p* = 0.11; HR = 1.23 (95%CI 0.74–2.06)), or OS (*p* = 0.36; HR = 1.23 (95%CI 0.58–2.61)). Though no significant impact was observed in OS, yet *BRCA1/2* mutation carriers were at high risk of recurrence, highlighting the importance of adopting *BRCA* screening strategies and prophylactic measures.

## 1. Introduction

Breast cancer is the most commonly occurring cancer in females and the leading cause of global cancer-related mortality [1,2]. Its marked impact is shifting gradually to the developing world and may even exceed that of Western industrialized societies in the near future [3,4]. These countries of emerging economies suffer from high incidence of breast cancer and advanced stage at diagnosis, coupled with limited health and medical care resources [5].

Molecular genetic studies have elucidated breast-cancer susceptibility genes 1 and 2 (*BRCA1* and *BRCA2*) as two major predisposing genes for breast cancer [6]. Inherited *BRCA1* and *BRCA2* mutations are associated with increased lifetime risks of breast and ovarian cancers by 45–75% and 18–40%, respectively [7,8,9,10], as well as other cancers like pancreatic and prostate cancers [11].

In addition to the established predictive importance of *BRCA1/2* mutation status in evaluating breast cancer risk [12,13], the identification of carriers of *BRCA1/2* mutations has significant implications in guiding surgical, radiotherapeutic, and drug treatment options [14,15]. Emerging research studies have demonstrated the clinical significance of *BRCA1/2* mutation status in predicting the response to chemotherapy [16] and poly(ADP-ribose) polymerase (PARP) inhibitors [17,18].

Moreover, *BRCA1/2* mutations have been studied as markers of pathological aggressiveness, with *BRCA1*-mutated tumors usually being of high histological grade at diagnosis, poorly differentiated, and triple negative, whereas *BRCA2*-related tumors are on average of high grade than non-carriers [19,20,21]. These unique histopathological features support the notion that *BRCA1/2* mutation carriers may have different prognosis in comparison to sporadic cases [22,23].

Nonetheless, the prognostic significance of *BRCA1/2* mutational status on breast cancer survival is still debatable. Few published clinical studies have found that breast cancer patients with *BRCA1/2* mutations show better prognosis than control groups [24,25], while others have reported that they have worse survival outcomes [26,27,28], whereas some studies reported similar prognosis [29,30,31,32,33]. This disparity might be attributed to discrepancies in methodological issues (including study size, design, studied populations), relatively low incidence of the *BRCA1/2* mutations, lack of adjustments for clinical variables, including risk-reducing options or treatment strategies, and short follow-up.

Previous studies have shown possible molecular, clinical, and epidemiological differences in breast cancer worldwide [34,35,36]. Though the incidence and profile of *BRCA1/2* mutations is population specific, data on clinical outcomes in different populations are scarce and most of the conducted studies included data on white patients, thereby potentially underestimating differences pertinent to genetic defects. These data highlight the need to elucidate the effect of *BRCA1/2* mutations on breast cancer prognosis in different populations, as this can impact future risk assessment and treatment planning. To our knowledge, sparse clinical data have been published in the Middle East to substantiate this assertion.

We previously reported the profile of *BRCA1/2* mutations in a cohort of 103 Egyptian female breast cancer patients who were not selected on the basis of age at onset of breast cancer or family history [37]. This study was conducted to elucidate the clinicopathological characteristics and the prognostic relevance of *BRCA1*/*2* mutations on long-term survival outcome in Egyptian female breast cancer patients. These findings will help to improve treatment options and surveillance policies for breast cancer patients harboring *BRCA1/2* mutations.

## 2. Materials and Methods

### 2.1. Data Collection and Ethical Statement

A cohort of 103 Egyptian female patients diagnosed with primary invasive breast cancer were retrospectively included in the study. The study was conducted according to the guidelines of the Declaration of Helsinki and approved by the Ethical Committee of Ain Shams University, Egypt.

Clinicopathological and demographic data were extracted from hospital medical records including age at primary breast cancer diagnosis, age at menarche, menopausal status at diagnosis, marital status, parity, the use of hormonal contraception, age at first full-term pregnancy and nursing. Family history of cancer and familial relationships between family members with cancer was collected by questionnaire. Tumor characteristics with regard to pathological stage, tumor size, nodal involvement, evidence of metastasis, histological type, estrogen receptor (ER), progesterone receptor (PR) and human epidermal growth factor receptor (HER-2) status, and type of surgery performed were collected from the pathology and medical records.

Follow-up data regarding date of diagnosis, clinical treatment details, the course of the disease, as well as site of disease progression (recurrence or metastatic disease), if any, were retrieved from medical records until death or loss to follow-up. The date of last follow-up assessment was retrieved from the medical records and through the treating physician, via telephone contact with the patient or her next-of-kin.

### 2.2. Mutational Analysis

*BRCA1/2* mutations were tested using HRM analysis and direct sequencing as previously described [37]. DNA was extracted from whole blood samples collected at the time of patient recruitment. The detected variants were classified according to the American College of Medical Genetics and Genomics and the Association for Molecular Pathology (ACMG/AMP) guidelines.

### 2.3. Outcomes Measures

Patients were followed from the date they were diagnosed with primary invasive breast cancer until the last follow-up date (the last date at which the patients were seen alive or free of the disease) or until death. The OS was calculated as the time from date of diagnosis until the date of death from any cause or last follow up. The RFS was defined as the time from date of surgery until the date of any loco-regional recurrence, contralateral breast tumor, death, or last follow-up. The DFS was determined as the time from date of surgery until the date of recurrence, distant metastasis, death, or last follow-up. The MFS was defined as time from date of surgery until the date of any distant metastasis, death, or last follow-up.

### 2.4. Statistical Analysis 

The clinicopathologic characteristics were compared according to *BRCA* mutation status using Chi-squared test for categorical variables and *t* test for continuous variables. Multivariate logistic regression was used to determine the factors predictive of *BRCA1*/*2* mutations. The log-rank test was used to perform comparisons between groups. The independent association of mutation status with outcome was determined using Cox proportional hazards regression analysis. Hazard ratios (HR) and their 95% confidence intervals (CI) for multivariable analyses were estimated. A *p* ≤ 0.05 was considered statistically significant. Statistical analysis was performed using IBM© SPSS© Statistics version 26 (IBM© Corp., Armonk, NY, USA).

## 3. Results

### 3.1. Demographic and Clinicopathological Characteristics of the Study Cohort 

In total, *BRCA1/2* variants were detected in 46 patients; 29 patients were carriers of *BRCA1/2* pathogenic variants (ACMG/AMG Class 5). The profile of the detected mutations was previously reported [37]. The majority of patients (91.3%) were treated with modified radical mastectomy (MRM). More than 70% of cases have received adjuvant chemotherapy: cyclophosphamide, methotrexate, and fluorouracil (CMF) regimen; or flourouracil, adriamycin, cyclophosphamide (FAC) regimen. In addition, 71.8% of cases have received hormonal therapy. The clinicopathological characteristics of the cohort are shown in Table 1. 

Median ages at diagnosis were 40 years (range, 24–57 years) in *BRCA1/2* carriers of pathogenic variants and 48 years (range, 39–66 years) in *BRCA* non-carriers (*p* = 0.008). In comparison to non-carriers, *BRCA1/2* carriers were more likely to have early onset breast cancer (48% versus 28%, *p* = 0.05), to be premenopausal at the time of diagnosis (82% vs. 54%, *p* = 0.02), and to have family history of breast cancer (45% vs. 35%, *p* = 0.024). *BRCA1/2* carriers were also more likely to have positive family history of any cancer (OR: 3.969, 95%CI (1.623–9.71), *p* = 0.002). There was no statistically significant difference with respect to the median ages of menarche, first full-term pregnancy, parity, nursing, or the use of hormonal contraception. Positive lymph node metastasis was more frequently observed in *BRCA1/2* carriers (76% vs. 53%, *p* = 0.03). No statistical significance was observed in tumor size. The majority of patients had grade II invasive ductal carcinoma. The incidence of ER-negative, PR-negative and HER-2 negative tumors was higher in *BRCA1/2* carriers versus non carriers (28% vs. 22%, 38% vs. 30%, 76% vs. 67%, respectively) though not statistically significant. Multivariate logistic regression model in all carriers revealed that age at diagnosis (*p* =0.047), positive lymph node involvement (*p* = 0.05), family history of any cancer (*p* = 0.051, borderline) remained statistically independent predictors for *BRCA1*/2 mutations. More than 70% of cases have received adjuvant chemotherapy: cyclophosphamide, methotrexate, and fluorouracil (CMF) regimen; or flourouracil, adriamycin, cyclophosphamide (FAC) regimen. In addition, 71.8% of cases have received hormonal therapy.

When we compared *BRCA1* or *BRCA2* independently versus non-carriers, *BRCA1* carriers were found to be diagnosed at younger age than non-carriers (*p* = 0.006). Both *BRCA1* and *BRCA2* carriers tended to be premenopausal at time of diagnosis (*p* = 0.05). In comparison to non-carriers, 63% of *BRCA1* positive cases reported family history of breast cancer and 56% of *BRCA2* cases had family history of other cancers (*p* = 0.001). *BRCA2* carriers were more likely to have positive regional lymph node metastasis than non-carriers (*p* = 0.012). There was no significant difference regarding tumor size, histological type, grade, hormone receptor status or the presence of bilateral breast cancers between *BRCA1* or *BRCA2* carriers and non-carriers.

### 3.2. Prognosis of BRCA1/2 Mutation Carriers and Non-Carriers

#### 3.2.1. BRCA1/2 Carriers of Pathogenic Variants

**Figure 1:**
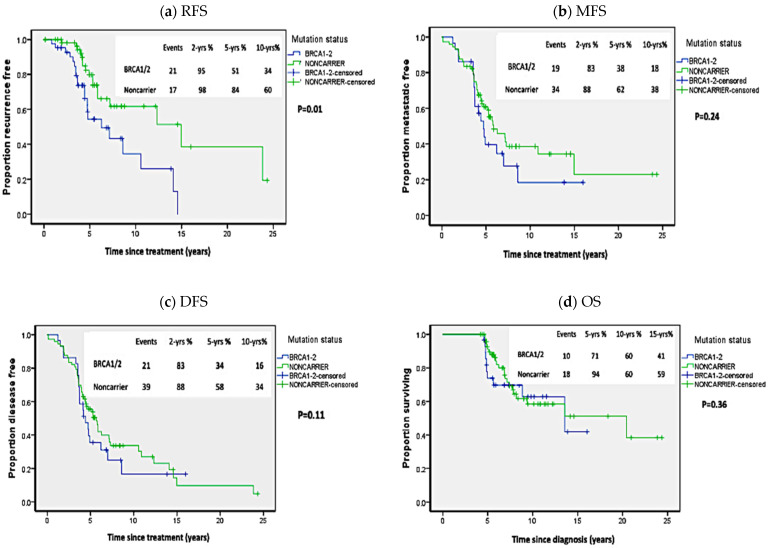
Kaplan Meier plots of survival analysis in carriers of *BRCA1/2* pathogenic mutations and non-carriers. (**a**) Recurrence-free survival (RFS), (**b**) Metastasis-free survival (MFS), (**c**) Disease-free survival (DFS), (**d**) Overall survival (OS). *p*-values calculated by log-rank test (considering whole follow-up period).

As shown in Figure 1, *BRCA1/2* carriers of pathogenic mutations exhibited significantly worse RFS in comparison to non-carriers (*p* = 0.01; HR = 3.00 (95%CI 1.35–6.68)). Though there was a trend toward worse MFS and DFS in *BRCA1/2* carriers compared to non-carriers, but the difference was not statistically significant (*p* = 0.24; HR = 1.38 (95%CI 0.8–2.4) and *p* = 0.11; HR = 1.23 (95%CI 0.74–2.06), respectively). Similarly, the difference in OS in *BRCA1/2* carriers versus non-carriers didn’t reach statistical significance (*p* = 0.36; HR = 1.23 (95%CI 0.58–2.61)). 

#### 3.2.2. All BRCA1/2 Mutation Carriers

**Figure 2:**
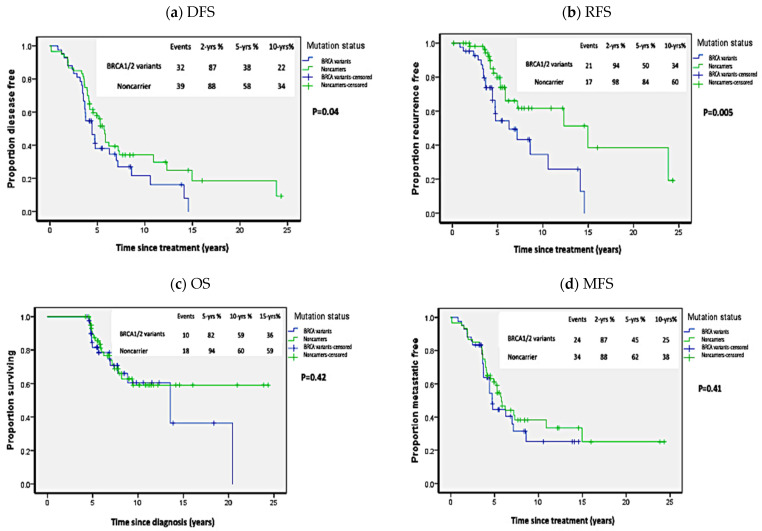
Kaplan Meier plots of survival analysis in carriers of all *BRCA1/2* variants. (**a**) Disease-free survival (DFS), (**b**) Recurrence-free survival (RFS), (**c**) Overall survival (OS), (**d**) Metastasis-free survival (MFS). *p*-values calculated by log-rank test (considering whole follow-up period).

When we compared the survival outcomes in carriers of all *BRCA1/2* variants to non-carriers, *BRCA1/2* carriers exhibited worse DFS than non-carriers: at two years 86% vs. 88%; at five years 38% vs. 58%; and at 10 years 22% vs. 34% (*p* = 0.04; HR = 2 [95%CI 1.1–2.4]) (Figure 2). As shown in Table 2, univariate analysis revealed that, in addition to *BRCA* mutation status, ER-, PR-negative receptor status, bilateral breast cancer, and large tumor size (T3–T4) were associated with worse DFS. However, stepwise cox proportional regression analysis showed that only ER-negative receptor (HR = 2.44 (95%CI = 1.33–4.47); *p* = 0.004) and large tumor size (HR = 2.19 (HR = 1.21–3.98); *p* = 0.01) were the only variables that independently affected DFS. The RFS was significantly worse in all carriers compared to non-carriers. The RFS at two years was 94%vs 98%; at five years: 50% vs. 84%; and at 10 years 34% vs. 60% (*p* = 0.005, HR = 2.53 (95%CI 1.3–4.92)). However, as shown in Figure 2, no statistical difference was noted in OS (*p* = 0.42; HR = 1.32 (95%CI 0.66–2.62)) or MFS (*p* = 0.41; HR = 1.25 (95%CI 0.74–2.11)), where OS was 82%, 59%, and 36% vs. 94%, 60%, and 59% at five, 10, and 15 years in carriers versus non-carriers, respectively. While MFS in all carriers versus non carriers was 87%, 45%, and 25% vs. 88%, 62%, and 38% at two, five, and 10 years, respectively.

#### 3.2.3. BRCA1 and BRCA2 Carriers Independently 

**Figure 3:**
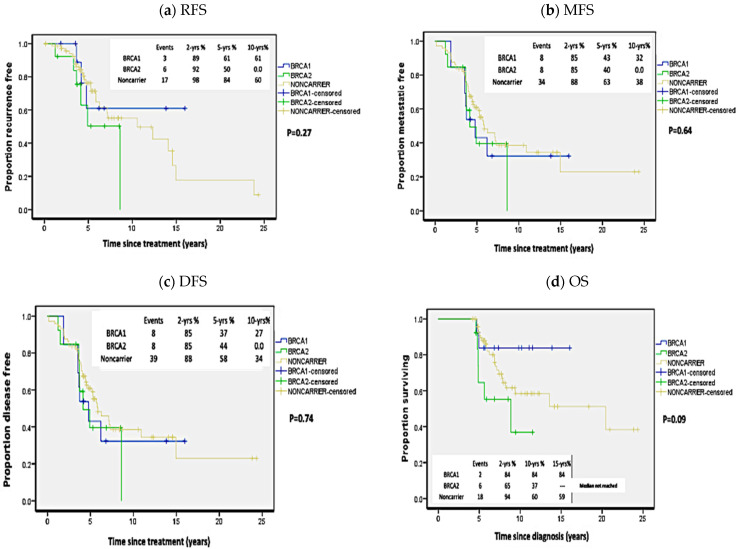
Kaplan Meier plots of survival analysis in carriers of *BRCA1* and *BRCA2* mutations independently and non-carriers. (**a**) Recurrence-free survival (RFS), (**b**) Metastasis-free survival (MFS), (**c**) Disease-free survival (DFS), (**d**) Overall survival (OS). *p*-values calculated by log-rank test (considering whole follow-up period).

No statistically significant difference was detected between carriers of *BRCA1* or *BRCA2* pathogenic mutations when analyzed independently with regards to RFS (*p* = 0.27; HR = 1.71 (95%CI 0.79–3.7); MFS (*p* = 0.64; HR = 1.38 (95%CI 0.78–2.44)); DFS (*p* = 0.74; HR = 1.38 (95%CI 0.8–2.36)) or OS (*p =* 0.09; HR = 1.36, (95%CI 0.62–2.98)) (Figure 3).

#### 3.2.4. BRCA1 Mutation c.5205delA

As previously reported [37], this pathogenic mutation was detected in high frequency in our cohort. Hence, we sought to analyze the clinicopathological characteristics and survival outcome in the patients harboring this mutation in comparison to non-carriers. However, no significant difference was detected except for young age at diagnosis (*p* = 0.036). The association of this mutation with OS, DFS, RFS, or DFS did not reach statistical significance (Table 3).

## 4. Discussion

Besides their impact on the susceptibility to breast cancer, *BRCA1/2* mutations may be linked to distinctive clinical course and biological features. We sought to retrospectively evaluate the impact of the *BRCA1/2* variants that were previously detected in this cohort, on the long-term survival outcomes over 24 years (1997–2019). 

In this population-based retrospective study, the median follow-up period was 6.9 years (range, 4.2–24.4 years). *BRCA1/2* carriers exhibited significantly worse RFS than non-carriers, with three-fold increased risk of contralateral breast cancer or locoregional recurrence. This finding is in broad agreement with results of other studies. For instance, Verhoog et al. showed that the development of contralateral breast cancer was 4–5 times more frequent in *BRCA1* mutation carriers than in the sporadic patients [38]. Bordeleau and colleagues also reported a 10-year increased risk of contralateral breast cancers of 20–40% in *BRCA* mutation carriers [31]. By the age of 70 years, *BRCA1* and *BRCA2* carriers were previously found to have average cumulative risk of contralateral breast cancer of 83% and 62%, respectively [10]. Ye et al. also showed higher risk of contralateral breast cancer in *BRCA* mutation carriers [39].

Nonetheless, no difference in DFS was noted between carriers of pathogenic variants in this study and non-carriers, although carriers of all *BRCA1/2* variants showed slightly worse DFS that could not independently predict survival. A recent study by Vocka et al. [40] recently reported slightly worse DFS in carriers of *BRCA1/2* mutations, whereas a meta-analysis by van den Broek et al. [26] showed that *BRCA1/BRCA2* mutation carriers had a non-significant tendency towards poorer survival.

Furthermore, our results did not reveal a statistically significant difference between *BRCA1/2* mutation carriers and non-carriers regarding MFS or OS. Some studies showed worse OS in patients with *BRCA1* mutations compared to sporadic cases [41,42]. Schmidt et al. reported that *BRCA1/2* mutation carriers who were diagnosed with breast cancer before the age of 50 years had worse OS that may be attributed to differences in tumor features, response to treatment, and secondary cancers [28]. Similarly, *BRCA1* mutation only was previously shown to decrease the OS and progression-free survival [43]. Besides, a metanalysis by Barretta and colleagues, including 105,220 breast cancer patients from 60 studies, showed worse OS and worse breast cancer specific survival (BCSS) in *BRCA1* mutation carriers while *BRCA2* carriers had worse BCSS only in comparison to sporadic cases [27]. However, other reports revealed similar survival outcome in both mutation carriers and non-carriers [31,38,44,45,46,47]. Systematic reviews with meta-analysis of survival outcome in *BRCA1* or *BRCA2* mutation carriers and non-carriers showed that current evidence did not support either poorer or better survival of *BRCA* carriers [26,27,32]. The POSH prospective study also found no significant differences in OS or distant DFS in patients with and without *BRCA1* or *BRCA2* mutations [48]. 

*BRCA*-associated breast cancers have unique clinicopathological characteristics compared to sporadic counterparts. In agreement with previous studies [22,41,49,50], carriers of *BRCA1/2* mutations in the current study are characterized by early onset, positive family history of breast cancer, premenopausal state at diagnosis, and positive lymph node involvement. Other reports showed that *BRCA*-associated tumours are more often ER and PR negative [51,52]. However, though *BRCA1/2* mutation carriers in the current study showed higher incidence of negative hormone receptors status than non-carriers, the difference was statistically indistinguishable. Consistent with prior observation [22], HER-2 expression status was similar in patients with and without mutations. Our findings show that early age at diagnosis, lymph node metastasis, and positive family history of any cancer are independent predictors *BRCA1/2* mutations, which have important clinical implications for screening and early diagnosis in our population.

As previously reported by Kwong et al. [50], *BRCA1* carriers were found to be younger at the time of diagnosis than non-carriers. *BRCA2* carriers showed positive family history of other cancers, which may indicate that in addition to breast cancer, *BRCA2* mutations may increase the risk of developing other cancers including prostate, pancreatic, pharyngeal, brain cancers and leukemia [53]. Unlike other studies illustrating that *BRCA1* mutant tumors feature higher grade, invasive borders and higher proliferation indices; while *BRCA2* mutation carriers are more likely to present with ER positive tumors and increased risk of contralateral breast cancer [29,44], however these differences were not noted in the current study. Additionally, we couldn’t demonstrate distinct survival outcomes when assessing the impact of *BRCA1* and *BRCA2* mutations separately. This finding is in line with the work of Templeton et al., a systematic review and meta-regression study that included 10,180 patients from 16 studies, showing that *BRCA1* or *BRCA2* mutations independently were not associated with worse OS [33]. 

The *BRCA1* pathogenic mutation c.5205delA was a novel mutation detected for the first time in our cohort (NCBI ClinVar VCV000140168). Patients harboring this mutation were diagnosed at significantly younger age than non-carriers. However, this mutation was not linked to worse survival outcomes. 

*BRCA* mutation status could provide important insights regarding prevention, surveillance, and treatment strategies [15]. Primary prevention measures, including prophylactic mastectomy, chemoprevention, and intensive surveillance, can decrease the risk of breast cancer in *BRCA1*/*BRCA2* mutations [54,55,56]. The standard treatment protocols for *BRCA* mutation carriers are still debatable. Studies have shown that contralateral mastectomy in breast cancer patients with *BRCA1/2* mutations can decrease breast cancer specific mortality [57]. A study including 1504 patients with germline *BRCA1* or *BRCA2* mutations showed that chemoprevention using tamoxifen is associated with a 50% reduction in the risk for developing contralateral breast cancer [45]. In addition, 10 years of tamoxifen therapy can also reduce the risk of breast cancer recurrence in premenopausal women [58]. Some studies have also shown that *BRCA1/2* mutation carriers exhibit different response to chemotherapy [59,60]. Under the precision medicine initiative, tailored treatment strategies including PARP inhibitors can be beneficial to carriers of *BRCA1* and *BRCA2* mutations [17,18]. 

The results of this study may unveil the underlying genetic alterations that may be predominating in the disparate population of Egyptian female breast cancer patients that may be linked to clinical characteristics and outcome. Major strengths of the current study are the long follow-up time and the unselected cohort. Nevertheless, study limitations lay in the retrospective study design as the 24-year study period (1997–2019) should have witnessed changes in diagnostic modalities and treatment options. Another limitation is the limited sample size. However, the meta-analysis by Baretta et al. on breast cancer survival in carriers of *BRCA1/BRCA2* mutations included 60 studies with the number of mutation carriers ranging from 5 to 326 (median, 39.5) [27]. Further prospective studies on larger cohorts are warranted to ascertain the prognostic significance and clinicopathological differences in *BRCA1/2* mutation carriers, aiming to optimize treatment choices and surveillance policies in patients harboring these mutations.

## 5. Conclusions

To the best of our knowledge, this is the first study in the Middle East to report long-term survival outcome of *BRCA1/2* related breast cancer. Based on our findings, *BRCA*-associated breast cancers showed a high pattern of locoregional recurrence and contralateral breast cancer. Herein, we emphasize the importance of adopting *BRCA* screening strategies and patient counselling regarding prophylactic measures and tailored treatment options in the mainstream oncology practice in Egypt.

## Figures and Tables

**Table 1:** Clinicopathological characteristics of *BRCA1/BRCA2* mutation carriers (together and separately) and non-carriers.

Factors	BRCA1/2 (*n* = 29)		BRCA1 (*n* = 16)		BRCA2 (*n* = 16)		All BRCA1/2 Variants (*n* = 46)		No BRCA Mutation (*n* = 57)
	N (%)	*p*	N (%)	*p*	N (%)	*p*	N (%)	*p*	N (%)
**Age at diagnosis**									
≤40	14 (48)	**0.05**	9 (56)	**0.024**	7 (44)	0.17	27 (59)	**0.002**	16 (28)
>40	15 (52)		7 (44)		9 (56)		19 (41)		41 (72)
**Median age at diagnosis**									
Year	40	**0.008**	40	**0.006**	41	0.26	40	**0.002**	48
(range)	(24–57)		(24–61)		(39–66)		(24–63)		(39–66)
**Family history of cancer**									
Breast cancer									
Other cancers	13 (45)	**0.024**	10 (63)	0.125	5 (31)	**0.001**	21 (46)	**0.005**	20 (35)
No	10 (34)		2 (12)		9 (56)		16 (34)		9 (15)
	6 (21)		4 (25)		2 (12)		9 (20)		28 (50)
**Menopausal state**									
Premenopausal	23 (79)	**0.02**	13 (81)	**0.05**	13 (81)	**0.05**	37 (80)	**0.006**	31 (54)
Postmenopausal	6 (21)		3 (19)		3 (19)		9 (20)		26 (46)
**Tumor size**									
T1–T2	20 (69)	0.7	13 (81)	0.45	9 (56)	0.2	27 (59)	**0.02**	41 (72)
T3–T4	9 (31)		3 (19)		7 (44)		19 (41)		16 (28)
**Lymph node involvement**									
Negative									
positive	7 (24)	**0.03**	10 (62)	0.4	2 (12)	**0.012**	10 (22)	**0.007**	27 (47)
	22 (76)		6 (38)		14 (88)		36 (78)		30 (53)
**Histological grade**									
G1	0		0		0		0		0
G2	28 (96.6)	0.62	15 (94)	0.33	16 (100)	0.59	44 (97)	0.35	56 (98)
G3	1 (3.4)		1 (6)		0 (0)		2 (3)		1 (2)
**Histological type**									
Ductal	25 (86.2)		13 (81)		15 (94)		42 (91)		54 (95)
Lobular	3 (10.3)	0.24	2 (13)	0.45	1 (6)	0.9	3 (7)	0.5	3 (5)
Mixed	1 (3.5)		1 (6)		0		1 (2)		0
**ER status**									
Positive	21 (72)	0.49	12 (75)	0.85	10 (63)	0.23	30 (65)	0.17	45 (78)
Negative	8 (28)		4 (25)		6 (37)		16 (35)		12 (22)
**PR status**									
Positive	18 (62)	0.44	11 (69)	0.9	8 (50)	0.13	26 (57)	0.15	40 (70)
Negative	11 (38)		5 (31)		8 (50)		20 (43)		17 (30)
**HER2 status**									
Positive	6 (24)	0.29	3 (23)	0.5	4 (29)	0.4	14 (35)	0.2	14 (33)
Negative	19 (76)		10 (77)		10 (71)		26 (65)		28 (67)
Not available	4		3		2		6		15
**Laterality**									
Unilateral	24 (83)	0.2	14 (88)	0.25	13 (81)	0.25	35 (76)	**0.007**	52 (91)
Bilateral	5 (17)		2 (12)		3 (19)		11 (24)		5 (9)

T, tumor size; G, grade; ER, estrogen receptor; PR, progesterone receptor; Her-2, human epidermal growth factor receptor 2; Bold, statistically significant differences as compared to *BRCA1/2* non-carriers.

**Table 2:** Univariate analysis for disease-free survival (DFS).

Factors		Number of Cases	Number of Events	DFS % 1 Year	Median	*p*-Value
**Age, years**	≤40	43	71	24.3	4.9	0.41
	>40	60	33	23.8	4.7	
**Menopause**	Post	35	38	24.4	5.3	0.16
	Pre	68	20	42.2	5.3	
**Nursing**	No	35	51	18.8	4.7	0.68
	Yes	68	23	18.8	5.3	
**Hormonal contraceptive**	No	36	48	26.0	4.7	0.83
	Yes	67	25	34.6	5.7	
**ER**	Negative	29	43	18.3	5.3	**0.02**
	Positive	74	22	18.3	3.9	
**PR**	Negative	37	49	27.7	5.8	**0.03**
	Positive	66	29	20.8	4.4	
**HER-2**	Negative	53	42	31.4	5.8	0.80
	Positive	29	38	6.8	4.5	
**Lymph node**	N0	37	20	0.0	4.0	0.74
	N1	66	25	26.1	5.3	
**T-stage**	T1-T2	68	46	22.9	5.0	**0.012**
	T3-T4	35	22	45.7	30.5	
**Laterality**	Bilateral	16	16	25.0	12.5	**<0.001**
	Unilateral	87	29	65.5	65.5	
**Family history**	No	40	23	29.4	5.7	0.29
	Yes	63	47	20.0	4.4	
**BRCA1/*2***	Carrier	46	32	13.2	4.2	**0.024**
	Non-carrier	57	39	31.5	5.8	

Bold: statistically significant.

**Table 3:** Survival outcomes in patients harboring *BRCA1* mutation c.5205delA (*p*.Val1736Serfs*29) as compared to non-carriers.

Outcome	Mutation Status	No. of Cases	No.of Events	%2 yrs	%5 yrs	%10 yrs	*p*-Value
RFS	Mut carrier	16	6	100	67.7	60.2	0.740
	Noncarrier	57	17	98.2	84.0	60.1	
MFS	Mut carrier	16	11	100	42.2	28.1	0.462
	Noncarrier	57	34	87.7	62.7	38.4	
DFS	Mut carrier	16	12	100.0	42.2	21.1	0.403
	Noncarrier	57	37	87.7	64.6	34.0	
OS	Mut carrier	16	4	100.0	86.7	75.8	0.582
	Noncarrier	57	18	100.0	94.0	60.6	

RFS: Recurrence-free survival, MFS: Metastasis-free survival, DFS: Disease-free survival, OS: Overall survival.

## Data Availability

Not applicable.

## References

[1] Siegel R.L., Miller K.D., Jemal A. (2019). Cancer statistics, 2019. CA Cancer J. Clin..

[2] Gong Y., Ji P., Hu X., Shao Z.-M. Abstract P2-08-16: The burden and trends of breast cancer from 1990 to 2017 at the global, regional, and national level: Results from the global burden of disease study 2017. Proceedings of the 2019 San Antonio Breast Cancer Symposium.

[3] Li N., Deng Y., Zhou L., Tian T., Yang S., Wu Y., Zheng Y., Zhai Z., Hao Q., Song D. (2019). Global burden of breast cancer and attributable risk factors in 195 countries and territories, from 1990 to 2017: Results from the Global Burden of Disease Study 2017. J. Hematol. Oncol..

[4] Winters S., Martin C., Murphy D., Shokar N.K., Lakshmanaswamy R. (2017). Breast Cancer Epidemiology, Prevention, and Screening. Approaches to Understanding Breast Cancer. Progress in Molecular Biology and Translational Science.

[5] Tfayli A., Temraz S., Abou Mrad R., Shamseddine A. (2010). Breast Cancer in Low- and Middle-Income Countries: An Emerging and Challenging Epidemic. J. Oncol..

[6] Szabo C.I., King M.C. (1997). Population genetics of BRCA1 and BRCA2. Am. J. Hum. Genet..

[7] Kuchenbaecker K.B., Hopper J.L., Barnes D.R., Phillips K.A., Mooij T.M., Roos-Blom M.J., Jervis S., van Leeuwen F.E., Milne R.L., Andrieu N. (2017). Risks of Breast, Ovarian, and Contralateral Breast Cancer for BRCA1 and BRCA2 Mutation Carriers. JAMA J. Am. Med. Assoc..

[8] Antoniou A., Pharoah P.D.P., Narod S., Risch H.A., Eyfjord J.E., Hopper J.L., Loman N., Olsson H., Johannsson O., Borg A. (2003). Average risks of breast and ovarian cancer associated with BRCA1 or BRCA2 mutations detected in case series unselected for family history: A combined analysis of 22 studies. Am. J. Hum. Genet..

[9] Chen S.N., Parmigiani G. (2007). Meta-analysis of BRCA1 and BRCA2 penetrance. J. Clin. Oncol..

[10] Mavaddat N., Peock S., Frost D., Ellis S., Platte R., Fineberg E., Evans D.G., Izatt L., Eeles R.A., Adlard J. (2013). Cancer Risks for BRCA1 and BRCA2 Mutation Carriers: Results From Prospective Analysis of EMBRACE. J. Natl. Cancer Inst..

[11] Cavanagh H., Rogers K.M.A. (2015). The role of BRCA1 and BRCA2 mutations in prostate, pancreatic and stomach cancers. Hered. Cancer Clin. Pract..

[12] Kwon J.S., Gutierrez-Barrera A.M., Young D., Sun C.C., Daniels M.S., Lu K.H., Arun B. (2010). Expanding the Criteria for BRCA Mutation Testing in Breast Cancer Survivors. J. Clin. Oncol..

[13] Begg C.B., Haile R.W., Borg A., Malone K.E., Concannon P., Thomas D.C., Langholz B., Bernstein L., Olsen J.H., Lynch C.F. (2008). Variation of breast cancer risk among BRCA1/2 carriers. JAMA J. Am. Med. Assoc..

[14] Trainer A.H., Lewis C.R., Tucker K., Meiser B., Friedlander M., Ward R.L. (2010). The role of BRCA mutation testing in determining breast cancer therapy. Nat. Rev. Clin. Oncol..

[15] Tung N.M., Garber J.E. (2018). BRCA1/2 testing: Therapeutic implications for breast cancer management. Br. J. Cancer.

[16] Caramelo O., Silva C., Caramelo F., Frutuoso C., Almeida-Santos T. (2019). The effect of neoadjuvant platinum-based chemotherapy in BRCA mutated triple negative breast cancers -systematic review and meta-analysis. Hered. Cancer Clin. Pract..

[17] Lee J.M., Ledermann J.A., Kohn E.C. (2014). PARP Inhibitors for BRCA1/2 mutation-associated and BRCA-like malignancies. Ann. Oncol..

[18] Kaufman B., Shapira-Frommer R., Schmutzler R.K., Audeh M.W., Friedlander M., Balmana J., Mitchell G., Fried G., Stemmer S.M., Hubert A. (2015). Olaparib Monotherapy in Patients with Advanced Cancer and a Germline BRCA1/2 Mutation. J. Clin. Oncol..

[19] Lakhani S.R., Jacquemier J., Sloane J.P., Gusterson B.A., Anderson T.J., van de Vijver M.J., Farid L.M., Venter D., Antoniou A., Storfer-Isser A. (1998). Multifactorial analysis of differences between sporadic breast cancers and cancers involving BRCA1 and BRCA2 mutations. J. Natl. Cancer Inst..

[20] Honrado E., Osorio A., Palacios J., Benitez J. (2006). Pathology and gene expression of hereditary breast tumors associated with BRCA1, BRCA2 and CHEK2 gene mutations. Oncogene.

[21] Southey M.C., Ramus S.J., Dowty J.G., Smith L.D., Tesoriero A.A., Wong E.E.M., Dite G.S., Jenkins M.A., Byrnes G.B., Winship I. (2011). Morphological predictors of BRCA1 germline mutations in young women with breast cancer. Br. J. Cancer.

[22] Atchley D.P., Albarracin C.T., Lopez A., Valero V., Amos C.I., Gonzalez-Angulo A.M., Hortobagyi G.N., Arun B.K. (2008). Clinical and pathologic characteristics of patients with BRCA-positive and BRCA-negative breast cancer. J. Clin. Oncol..

[23] Mavaddat N., Barrowdale D., Andrulis I.L., Domchek S.M., Eccles D., Nevanlinna H., Ramus S.J., Spurdle A., Robson M., Sherman M. (2012). Pathology of Breast and Ovarian Cancers among BRCA1 and BRCA2 Mutation Carriers: Results from the Consortium of Investigators of Modifiers of BRCA1/2 (CIMBA). Cancer Epidemiol. Biomark. Prev..

[24] Maksimenko J., Irmejs A., Nakazawa-Miklasevica M., Melbarde-Gorkusa I., Trofimovics G., Gardovskis J., Miklasevics E. (2014). Prognostic role of BRCA1 mutation in patients with triple-negative breast cancer. Oncol. Lett..

[25] Cortesi L., Masini C., Cirilli C., Medici V., Marchi I., Cavazzini G., Pasini G., Turchetti D., Federico M. (2010). Favourable ten-year overall survival in a Caucasian population with high probability of hereditary breast cancer. BMC Cancer.

[26] van den Broek A.J., Schmidt M.K., van’t Veer L.J., Tollenaar R., van Leeuwen F.E. (2015). Worse Breast Cancer Prognosis of BRCA1/BRCA2 Mutation Carriers: What’s the Evidence? A Systematic Review with Meta-Analysis. PLoS ONE.

[27] Baretta Z., Mocellin S., Goldin E., Olopade O.I., Huo D. (2016). Effect of BRCA germline mutations on breast cancer prognosis: A systematic review and meta-analysis. Medicine.

[28] Schmidt M.K., van den Broek A.J., Tollenaar R., Smit V., Westenend P.J., Brinkhuis M., Oosterhuis W.J.W., Wesseling J., Janssen-Heijnen M.L., Jobsen J.J. (2017). Breast Cancer Survival of BRCA1/BRCA2 Mutation Carriers in a Hospital-Based Cohort of Young Women. JNCI J. Natl. Cancer Inst..

[29] Brekelmans C.T.M., Tilanus-Linthorst M.M.A., Seynaeve C., Van der Ouweland A., Menke-Pluymers M.B.E., Bartels C.C.M., Kriege M., van Geel A.N., Burger C.W., Eggermont A.M.M. (2007). Tumour characteristics, survival and prognostic factors of hereditary breast cancer from BRCA2-, BRCA1- and non-BRCA1/2 families as compared to sporadic breast cancer cases. Eur. J. Cancer.

[30] Rennert G., Bisland-Naggan S., Barnett-Griness O., Bar-Joseph N., Zhang S.Y., Rennert H.S., Narod S.A. (2007). Clinical outcomes of breast cancer in carriers of BRCA1 and BRCA2 mutations. N. Engl. J. Med..

[31] Bordeleau L., Panchal S., Goodwin P. (2010). Prognosis of BRCA-associated breast cancer: A summary of evidence. Breast Cancer Res. Treat..

[32] Zhong Q., Peng H.L., Zhao X., Zhang L., Hwang W.T. (2015). Effects of BRCA1- and BRCA2-Related Mutations on Ovarian and Breast Cancer Survival: A Meta-analysis. Clin. Cancer Res..

[33] Templeton A.J., Gonzalez L.D., Vera-Badillo F.E., Tibau A., Goldstein R., Seruga B., Srikanthan A., Pandiella A., Amir E., Ocana A. (2016). Interaction between Hormonal Receptor Status, Age and Survival in Patients with BRCA1/2 Germline Mutations: A Systematic Review and Meta-Regression. PLoS ONE.

[34] Momenimovahed Z., Salehiniya H. (2019). Epidemiological characteristics of and risk factors for breast cancer in the world. Breast Cancer Targets Ther..

[35] Sopik V. (2021). International variation in breast cancer incidence and mortality in young women. Breast Cancer Res. Treat..

[36] Hortobagyi G.N., de la Garza Salazar J., Pritchard K., Amadori D., Haidinger R., Hudis C.A., Khaled H., Liu M.C., Martin M., Namer M. (2005). ABREAST Investigators. The global breast cancer burden: Variations in epidemiology and survival. Clin. Breast Cancer.

[37] AbdelHamid S.G., Zekri A.N., AbdelAziz H.M., El-Mesallamy H.O. (2021). BRCA1 and BRCA2 truncating mutations and variants of unknown significance in Egyptian female breast cancer patients. Clin. Chim. Acta.

[38] Verhoog L.C., Brekelmans C.T.M., Seynaeve C., van den Bosch L.M.C., Dahmen G., van Geel A.N., Tilanus-Linthorst M.M.A., Bartels C.C.M., Wagner A., van den Ouweland A. (1998). Survival and tumour characteristics of breast-cancer patients with germline mutations of BRCA1. Lancet.

[39] Ye F.G., Huang L., Lang G.T., Hu X., Di G.H., Shao Z.M., Cao A.Y. (2020). Outcomes and risk of subsequent breast events in breast-conserving surgery patients with BRCA1 and BRCA2 mutation. Cancer Med..

[40] Vocka M., Zimovjanova M., Bielcikova Z., Tesarova P., Petruzelka L., Mateju M., Krizova L., Kotlas J., Soukupova J., Janatova M. (2019). Estrogen Receptor Status Oppositely Modifies Breast Cancer Prognosis in BRCA1/BRCA2 Mutation Carriers Versus Non-Carriers. Cancers.

[41] Wang Y.A., Jian J.W., Hung C.F., Peng H.P., Yang C.F., Cheng H.C.S., Yang A.S. (2018). Germline breast cancer susceptibility gene mutations and breast cancer outcomes. BMC Cancer.

[42] Stoppa-Lyonnet D., Ansquer Y., Dreyfus H., Gautier C., Gauthier-Villars M., Bourstyn E., Clough K.B., Magdelenat H., Pouillart P., Vincent-Salomon A. (2000). Familial invasive breast cancers: Worse outcome related to BRCA1 mutations. J. Clin. Oncol..

[43] Lee E.H., Park S.K., Park B., Kim S.W., Lee M.H., Ahn S.H., Son B.H., Yoo K.Y., Kang D., Grp K.R. (2010). Effect of BRCA1/2 mutation on short-term and long-term breast cancer survival: A systematic review and meta-analysis. Breast Cancer Res. Treat..

[44] Goodwin P.J., Phillips K.A., West D.W. (2007). Prognosis of breast cancer in carriers of BRCA1 and BRCA2 mutations. N. Engl. J. Med..

[45] Gronwald J., Robidoux A., Kim-Sing C., Tung N., Lynch H.T., Foulkes W.D., Manoukian S., Ainsworth P., Neuhausen S.L., Demsky R. (2014). Duration of tamoxifen use and the risk of contralateral breast cancer in BRCA1 and BRCA2 mutation carriers. Breast Cancer Res. Treat..

[46] Lee L.J., Alexander B., Schnitt S.J., Comander A., Gallagher B., Garber J.E., Tung N. (2011). Clinical Outcome of Triple Negative Breast Cancer in BRCA1 Mutation Carriers and Noncarriers. Cancer.

[47] Huzarski T., Byrski T., Gronwald J., Gorski B., Domagala P., Cybulski C., Oszurek O., Szwiec M., Gugala K., Stawicka M. (2013). Ten-Year Survival in Patients With BRCA1-Negative and BRCA1-Positive Breast Cancer. J. Clin. Oncol..

[48] Copson E.R., Maishman T.C., Tapper W.J., Cutress R.I., Greville-Heygate S., Altman D.G., Eccles B., Gerty S., Durcan L.T., Jones L. (2018). Germline BRCA mutation and outcome in young-onset breast cancer (POSH): A prospective cohort study. Lancet Oncol..

[49] Veronesi A., de Giacomi C., Magri M.D., Lombardi D., Zanetti M., Scuderi C., Dolcetti R., Viel A., Crivellari D., Bidoli E. (2005). Familial breast cancer: Characteristics and outcome of BRCA 1-2 positive and negative cases. BMC Cancer.

[50] Kwong A., Wong L.P., Wong H.N., Law F.B.F., Ng E.K.O., Tang Y.H., Chan W.K., Ho L.S., Kwan K.H., Poon M. (2009). A BRCA2 founder mutation and seven novel deleterious BRCA mutations in southern Chinese women with breast and ovarian cancer. Breast Cancer Res. Treat..

[51] Byrski T., Gronwald J., Huzarski T., Grzybowska E., Budryk M., Stawicka M., Mierzwa T., Szwiec M., Wisniowski R., Siolek M. (2008). Response to neo-adjuvant chemotherapy in women with BRCA1-positive breast cancers. Breast Cancer Res. Treat..

[52] Musolino A., Bella M.A., Bortesi B., Michiara M., Naldi N., Zanelli P., Capelletti M., Pezzuolo D., Camisa R., Savi M. (2007). BRCA mutations, molecular markers, and clinical variables in early-onset breast cancer: A population-based study. Breast.

[53] Roy R., Chun J., Powell S.N. (2012). BRCA1 and BRCA2: Different roles in a common pathway of genome protection. Nat. Rev. Cancer.

[54] Winship I., Southey M.C. (2016). Gene panel testing for hereditary breast cancer. Med. J. Aust..

[55] Rebbeck T.R., Friebel T., Lynch H.T., Neuhausen S.L., van’t Veer L., Garber J.E., Evans G.R., Narod S.A., Isaacs C., Matloff E. (2004). Bilateral prophylactic mastectomy reduces breast cancer risk in BRCA1 and BRCA2 mutation carriers: The PROSE study group. J. Clin. Oncol..

[56] Metcalfe K.A., Lubinski J., Ghadirian P., Lynch H., Kim-Sing C., Friedman E., Foulkes W.D., Domchek S., Ainsworth P., Isaacs C. (2008). Predictors of contralateral prophylactic mastectomy in women with a BRCA1 or BRCA2 mutation: The hereditary breast cancer clinical study group. J. Clin. Oncol..

[57] Metcalfe K., Gershman S., Ghadirian P., Lynch H.T., Snyder C., Tung N., Kim-Sing C., Eisen A., Foulkes W.D., Rosen B. (2014). Contralateral mastectomy and survival after breast cancer in carriers of BRCA1 and BRCA2 mutations: Retrospective analysis. Br. Med. J..

[58] Smith G.L. (2014). The Long and Short of Tamoxifen Therapy: A Review of the ATLAS Trial. J. Adv. Pract. Oncol..

[59] Chapppuis P.O., Goffin J., Wong N., Perret C., Ghadirian P., Tonin P.N., Foulkes W.D. (2002). A significant response to neoadjuvant chemotherapy in BRCA1/2 related breast cancer. J. Med. Genet..

[60] Kriege M., Seynaeve C., Meijers-Heijboer H., Collee J.M., Menke-Pluymers M.B.E., Bartels C.C.M., Tilanus-Linthorst M.M.A., Blom J., Huijskens E., Jager A. (2009). Sensitivity to First-Line Chemotherapy for Metastatic Breast Cancer in BRCA1 and BRCA2 Mutation Carriers. J. Clin. Oncol..

